# The protective role of self compassion in trauma recovery and its moderating impact on post traumatic symptoms and post traumatic growth

**DOI:** 10.1038/s41598-025-91819-x

**Published:** 2025-03-09

**Authors:** Marios Adonis, Marina Loucaides, Mark J. M. Sullman, Timo Lajunen

**Affiliations:** 1 Department of Social Sciences, School of Humanities and Social Sciences, University of Nicosia, Nicosia, Cyprus; 2 Department of Psychology, University of Helsinki, Helsinki, Finland; 3 Department of Psychology, Norwegian University of Science and Technology, Trondheim, Norway

**Keywords:** Trauma exposure, PTSD, PTS, Posttraumatic growth, Self-compassion, Posttraumatic stress, Psychology, Human behaviour

## Abstract

This study examined the moderating effect of self-compassion on the relationship between post-traumatic symptoms (PTS) and post-traumatic growth (PTG) among adults with trauma exposure. A sample of 413 participants (254 women, 155 men) aged 18 to 81 years (M = 33.8; SD = 12.9) completed questionnaires assessing trauma exposure, PTS, PTG, and self-compassion. The results indicated that women reported significantly higher PTS and lower self-compassion than men, while no significant gender differences were found for PTG. Correlational analyses revealed a significant positive association between PTS and PTG, and a significant negative association between PTS and self-compassion. Moderation analysis demonstrated that self-compassion significantly moderated the relationship between PTS and PTG, with higher levels of self-compassion linked to greater PTG, even at elevated levels of PTS. These findings underscore the importance of self-compassion as a protective factor in trauma recovery, promoting positive psychological transformation despite the presence of distress.

## Introduction

### Definition and impact of trauma

Exposure to trauma, whether a single incident, recurring events, or prolonged circumstances perceived as life-threatening or harmful^1,2^, can lead to diverse psychological outcomes. Trauma results from experiences or events that are overwhelming and involve significant threats to an individual’s emotional, physical, or psychological well-being and safety, leading to psychological distress and impairment^3^.

### PTSD, PTS, and PTG: understanding the spectrum of trauma responses

While frequently associated with adverse consequences such as post-traumatic stress disorder (PTSD), trauma can also catalyse positive psychological transformations, including post-traumatic growth (PTG)^4–7^. PTSD is a recognised clinical diagnosis that requires a set of specific criteria to be met. PTSD symptoms include intrusive thoughts, avoidance behaviours, negative alterations in cognition and mood, and marked alterations in arousal and reactivity that persist for more than a month and cause significant distress or impairment in functioning^8^. Post-traumatic growth (PTG) refers to the positive psychological change experienced as a result of the struggle with highly challenging life circumstances. This concept suggests that individuals can develop beyond their previous levels of functioning after encountering a traumatic event. PTG involves not merely a return to baseline, but rather an improvement in various areas of life^4,5,7^.

### Theoretical models of post-traumatic growth

Theoretical models of PTG emphasise the role of cognitive processing of the traumatic event, specifically meaning-making and cognitive reappraisal, as crucial mechanisms^7^. This stands in contrast to the distress and dysfunction characteristic of PTSD. Despite substantial research investigating the relationship between PTSD and PTG^9,10^, the factors influencing this complex interplay remain inadequately understood^11^.

According to Rutten et al.'s^12^ theoretical framework, individuals experience distinct trajectories of risk and resilience in psychopathology following trauma exposure. These trajectories can range from persistent mental health decline to an initial decline, followed by a recovery that surpasses pre-trauma well-being, a hallmark of PTG. PTG is believed to result from cognitive processing initiated when traumatic events disrupt existing worldviews and schemas^7^, with empirical evidence highlighting the critical role of positive reappraisal, acceptance, and deliberate meaning-making in fostering PTG^13–16^. Ultimately, PTG occurs when individuals successfully process their trauma, derive meaning from it, and cultivate a renewed and empowered perspective on life^12^. The pathway to PTG, however, is rarely straightforward. Trauma exposure can also precipitate PTSD symptoms, including intrusive thoughts, avoidance behaviours, negative mood alterations, and hyperarousal^17^, which can significantly impair daily functioning and interpersonal relationships. Importantly, PTSD does not necessarily preclude PTG. Some research suggests the two can co-occur, with PTSD potentially acting as a catalyst for transformative growth^4^.

While the idea of PTG as gaining strength through adversity is compelling and deeply rooted in humanistic psychology and cultural narratives, Infurna and Jayawickreme^18^ highlight the need for caution in interpreting PTG as evidence of profound psychological change. PTG could sometimes represent adaptive narratives individuals create to make sense of their trauma, without necessarily indicating deep internal change. Retrospective self-perceived measures of PTG may partly indicate meaningful personality change, but they can also encompass maladaptive reality distortions, selective appraisals, coping mechanisms, personality traits, interpretations of emotional states, reflections of implicit theories of change, and beliefs that one’s past self was worse than it truly was^18^. Despite these alternative interpretations of PTG, PTG remains an essential concept in trauma studies, as it offers valuable insights into how individuals navigate adversity, construct meaning, and find pathways toward resilience and recovery.

### Risk and resilience: factors influencing PTSD and PTG

Research has identified numerous individual and contextual factors influencing the development of both PTSD and PTG. Risk factors for PTSD include trauma severity, perceived threat, inadequate social support, and personality traits such as neuroticism^19^. Conversely, factors associated with greater PTG include engagement in active coping strategies, higher levels of subjective well-being, and personality traits such as openness to experience and extraversion^20–23^. Intriguingly, research suggests a potential curvilinear relationship between PTSD and PTG, with moderate PTSD levels associated with the highest levels of PTG, while both low and high levels correspond to lower PTG^4^.

### The role of self-compassion in trauma recovery

Within this complex interplay, self-compassion, a core construct within positive psychology^24,25^, has emerged as a potentially salient factor. Self-compassion, defined as a kind, understanding, and non-judgemental orientation towards oneself in the face of suffering, comprises three core components: self-kindness, common humanity, and mindfulness. A growing body of research has linked self-compassion to numerous adaptive outcomes, including reduced PTSD symptoms, lower levels of anxiety and depression, enhanced well-being, and the utilisation of more effective coping strategies^2,26–29^. Several studies have specifically demonstrated a negative relationship between PTSD and self-compassion^2,30–33^.

Higher levels of self-compassion appear to mitigate PTSD symptoms and facilitate PTG. Self-compassion may help individuals reframe their experience of suffering as part of the shared human experience, thereby potentially aiding in the reappraisal of the traumatic event and facilitating PTG^24,25,34^. Mindfulness, a key component of self-compassion, may promote calmer responses to traumatic memories and experiences, further correlating with higher PTG^35^.

While relatively few studies have explicitly investigated the tripartite relationship between self-compassion, PTSD, and PTG, preliminary findings are promising. Research suggests that self-compassion correlates with the adaptive cognitive processes associated with increased PTG^36^, mediates the relationship between trauma and PTG in bereaved parents^37^, and is positively correlated with distress tolerance and PTG^38^. Furthermore, self-compassion has been found to mediate the relationship between PTSD and PTG in students who experienced a natural disaster^39^.

As mentioned earlier, previous research has indicated a connection between self-compassion and positive outcomes. These outcomes include a reduction in symptoms of PTSD, decreased levels of anxiety and depression, improved well-being, and better coping skills. Furthermore, there seems to be a strong relationship or even overlap between self-compassion and neuroticism. In a study conducted by Pfattheicher and colleagues^40^, significant correlations were found between the neuroticism factor of the Five Factor Model of personality (FFM) and both the positive and negative aspects of self-compassion. The correlations between facets of neuroticism, such as Anxiety (r = 0.85), Depression (r = 0.90), and Self-consciousness (r = 0.85) from the NEO-PI-R, were so high that self-compassion and FFM neuroticism can be considered virtually identical constructs^40^. This suggests that while self-compassion may, in part, overlap with dimensions of neuroticism, its distinct framing and practical application remain invaluable for addressing trauma, mitigating post-traumatic symptoms, and fostering post-traumatic growth.

Earlier literature about the effects of traumatic evets and PTG focus mostly on PTSD. Not all people develop PTSD following exposure to trauma while still showing post-traumatic stress (PTS) symptoms such as anxiety, irritability, and sleep disturbances^33,41^. These PTS symptoms may resolve over time without intervention^41^. Furthermore, research has shown that recent PTS symptoms can predict future post-traumatic stress symptoms^42^. This indicates that, while PTS may not be as severe as PTSD, PTS symptoms can be seen as “subthreshold” PTSD, which can have lasting effects on individuals^8,42^. The present study focuses on PTS symptoms, which are naturally more common among trauma survivors than diagnosed PTSD^43^.

### Gender differences in trauma responses

Research has highlighted gender differences in psychological responses to trauma, including both PTSD and PTG. Women show a greater likelihood of developing PTSD after trauma, possibly explained by differences in the type of trauma experienced, coping mechanisms, and hormonal effects^44–47^. On the other hand, studies suggest that women also report higher levels of PTG compared to men, potentially reflecting greater engagement in meaning-making processes and social support networks^7,48^. This research emphasizes the complex interaction of biological, psychological, and sociocultural elements in influencing trauma responses based on gender. It is thus important to understand the gender differences in the development of PTSD, PTG as well as the role of self-compassion in order to design gender appropriate treatments.

### The current study

Above-described findings underscore the complex and nuanced interplay between the negative and positive outcomes of trauma and the importance of self-compassion in the trauma recovery process. While the independent relationships between self-compassion and both PTS and PTG have been explored, a critical gap remains in understanding the potential interplay between these constructs. Specifically, does self-compassion moderate the relationship between PTS and PTG, influencing the extent to which individuals experience positive growth even in the face of trauma-related distress? This study directly addresses this question by examining the moderating role of self-compassion on the relationship between PTS and PTG within a diverse sample of adults who have experienced trauma.

## Method

### Procedure

Sixty university student volunteers, selected through a convenience sampling method, distributed 600 questionnaire packets within their social networks, targeting a diverse range of age groups. Each packet contained four questionnaires designed to measure exposure to trauma, post-traumatic stress (PTS), post-traumatic growth (PTG), and self-compassion. Additionally, each packet included a consent form and detailed instructions. Participants were asked to sign the consent form before completing the questionnaires and returning the completed packets to the volunteers. The distribution was primarily conducted in person, with follow-up reminders to ensure a high return rate. The study received approval from the institutional research ethics committee, and all ethical guidelines, including those outlined in the Declaration of Helsinki, were strictly followed to protect participant confidentiality and well-being.

### Participants

Out of the 600 distributed packets, 494 (82%) were returned. The final sample consisted of 299 women (60.5%) and 189 men (38.3%), with ages ranging from 18 to 81 years old (M = 34.7, SD = 13.41). All participants were Greek-speaking Cypriots residing in the government-controlled areas of Cyprus. Educational backgrounds were varied: 26% had primary or secondary education, 48.6% had college or university education, 25% had post-graduate education, and one participant had no formal education. Regarding marital status, 38% were married, 51% were single, and 9.6% were divorced, widowed, or separated. Participants rated their socioeconomic status (SES) on a 10-point Likert scale (1 = worst possible SES, 10 = best possible SES), with a normal distribution (M = 5.94, SD = 1.36).

A priori power analysis was conducted using GPower (version 3.1.9.70 to calculate the necessary sample size for a multiple regression model. The model included three predictors and was set to detect a small effect size (f^2^ = 0.02) with a significance level of 0.05. The analysis determined that a sample size of 485 participants would be required to achieve a power of 0.80.

### Measures

#### Life Events Checklist for DSM-5 (LEC-5)

The LEC-5, or Life Events Checklist for DSM-5^49^, is a self-report questionnaire designed to collect information on potentially traumatic events in a person’s life. For this study, the questionnaire was translated into Greek. Participants specified whether each event occurred to them personally, to a close family member or friend, to someone they witnessed, as part of their job, or if they were unsure or it did not apply. The translated version was validated through a pilot study with 50 participants to ensure cultural relevance and comprehension. Participants reported that the items were clear and unambiguous, with no confusion about what was being asked.

#### PTSD Checklist, Civilian Version (PCL-C)

The PCL-C^50^ is a self-report instrument designed to measure post-traumatic stress symptoms. This study utilised the Greek version^51^. The 17-item scale employs a five-point severity scale (1 = not at all, 5 = extremely) to assess symptoms experienced over the past month. Total scores range from 17 to 85, with higher scores indicating more severe post-traumatic stress. The internal reliability of this measure, as indicated by Cronbach’s α, was 0.93. The validity of the Greek version has been confirmed through previous studies^51^.

#### Posttraumatic Growth Inventory (PTGI)

The PTGI^52^ is a self-report measure evaluating positive outcomes following trauma. This study used the Greek version^53^. The 21-item scale encompasses five domains: new possibilities, personal strength, spiritual change, relating to others, and appreciation of life. Participants were asked to reflect on their most traumatic event and to rate each of the 21 items on a six-point Likert scale, ranging from 0 (no change) to 5 (very great change). The internal reliability of this measure, indicated by Cronbach’s α, was 0.97. The validity of the Greek version has been established in prior research^53^.

#### Self-Compassion Scale (SCS)

The SCS^24,25^ is a self-report measure designed to assess self-compassion. This study utilized the Greek version^54^. The 26-item scale evaluates six elements: common humanity, self-kindness, self-judgment, mindfulness, over-identification, and isolation. The self-kindness subscale measures the ability to be kind and understanding toward oneself (e.g., “I try to be loving towards myself when I’m feeling emotional pain”), while the self-judgement subscale assesses the tendency to be critical of oneself (e.g., “I’m disapproving and judgemental about my own flaws and inadequacies”). Common humanity reflects the recognition that suffering and personal inadequacy are part of the shared human experience (e.g., “I try to see my failings as part of the human condition”), and isolation measures feelings of being alone in one’s struggles and failures (e.g., “When I think about my inadequacies, it tends to make me feel more separate and cut off from the rest of the world”). The mindfulness subscale evaluates the ability to hold one’s painful thoughts and feelings in balanced awareness (e.g., “When something painful happens, I try to take a balanced view of the situation”), while over-identification captures the tendency to become absorbed in negative emotions (e.g., “When I’m feeling down, I tend to obsess and fixate on everything that’s wrong”). Participants rate each item on a five-point Likert scale (1 = almost never, 5 = almost always), with higher scores indicating greater self-compassion. The internal reliability of this measure, as indicated by Cronbach’s α, was 0.86. The validity of the Greek version has been supported by previous validation studies^54^.

### Data handling and data analysis

All collected data were anonymised and securely stored in a password-protected database, accessible only to authorised researchers. The data were independently entered by two different research assistants. Any discrepancies identified were resolved by consulting the original questionnaires, ensuring complete accuracy of the data. Data analysis was conducted using version 26 of the Statistical Package for the Social Sciences (SPSS). Descriptive statistics and independent samples *t*-tests were performed to examine gender differences in study variables. Pearson correlations assessed the bivariate relationships between PTS, PTG, and self-compassion. To explore the moderating effect of self-compassion on the relationship between PTS and PTG, a moderation analysis was conducted using the PROCESS macro for SPSS^55^. PTS was entered as the independent variable, self-compassion as the moderator, and PTG as the dependent variable. All variables were mean centred prior to analysis to reduce multicollinearity.

## Results

### Descriptive statistics and gender differences

**Fig. 1:**
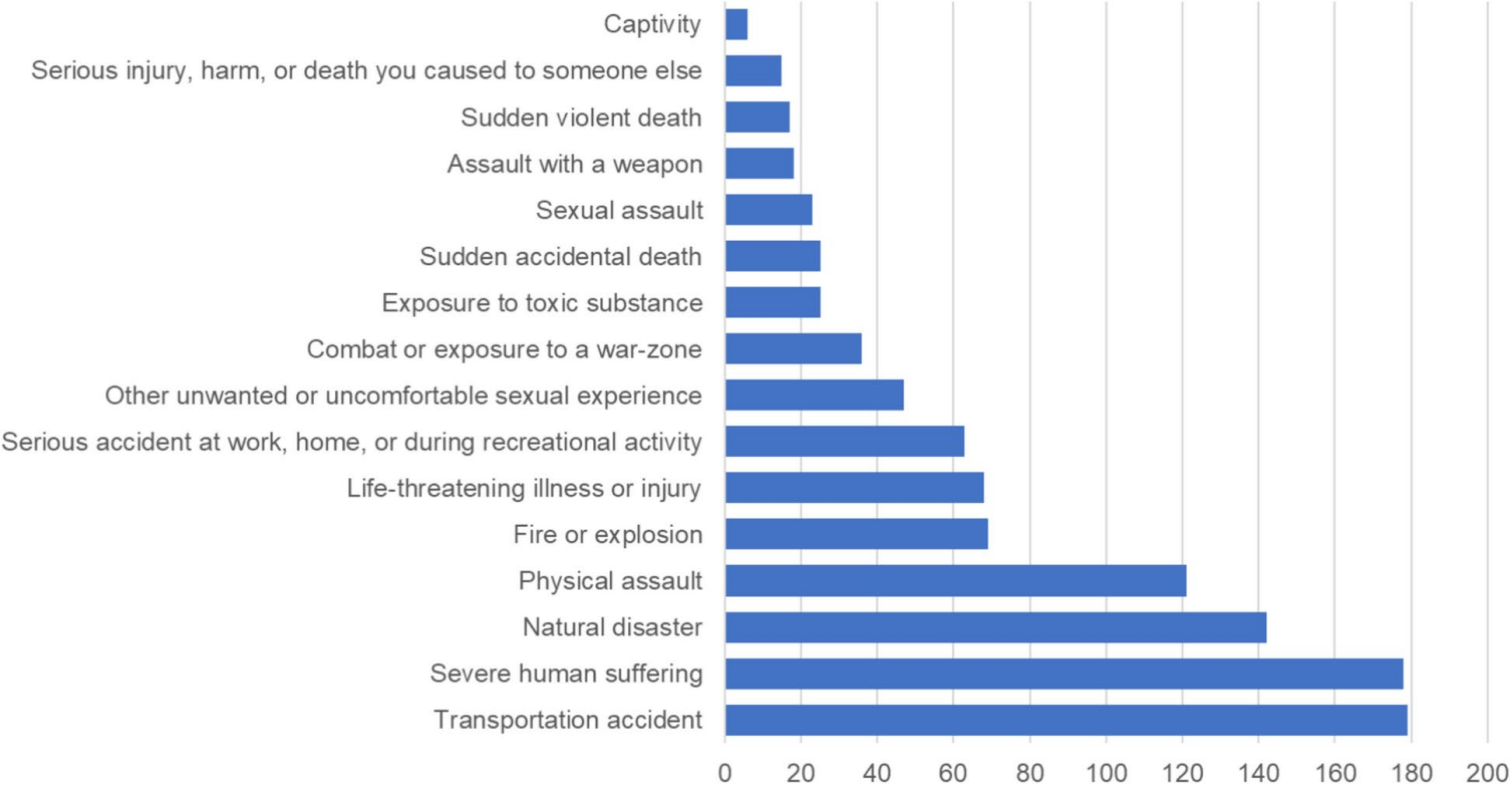
Number of participants reporting exposure to each type of trauma.

Among the 494 participants, 413 (84%) reported experiencing at least one traumatic event. Consequently, all subsequent analyses were performed exclusively on data from these individuals, with sample sizes ranging from 366 to 487 depending on responses to different instruments (Table 1). The most common trauma types were transportation accidents (36%), severe human suffering (36%), and natural disasters (29%; Fig. 1). Sixty-seven (13.6%) individuals reported sexual abuse, 141 (28.5%) reported physical abuse, and 346 (70%) reported situational trauma. Women were overrepresented for sexual abuse, and situational trauma, while men were overrepresented for physical abuse (Table 2).

**Table 1:** Descriptive statistics.

Variable	n	M	SD	Range
Post-traumatic symptoms	380	13.42	11.51	51
Post-traumatic growth	366	53.6	27.27	103
Self-compassion	466	82.29	14.93	92
Socioeconomic status	487	5.94	1.36	9

**Table 2:** Type of trauma exposure by gender.

Exposure	Women		Men	
	n	%	n	%
Situational trauma	215	71.91	125	66.14
Physical abuse	72	24.08	65	34.39
Sexual abuse	54	18.06	11	5.82

Independent samples *t*-tests indicated that women reported significantly higher PTS (t(481) = − 3.54, p < 0.001) and lower self-compassion (t(481) = 3.44, p < 0.001) compared to men. No significant gender differences emerged for PTG (Table 3).

**Table 3:** *T*-test results for sex-based differences.

	Sex	N	Mean	SD	t	p	Cohen’s d
Socioeconomic status	Female	296	5.85	1.33	*t*(483) = − 1.8	0.07	
	Male	189	6.08	1.40			
Post-traumatic symptoms	Female	268	14.34	12.11	*t*(433) = 2.07	0.04	0.18
	Male	167	11.91	11.52			
Post-traumatic growth	Female	257	54.32	28.15	*t*(412) = 0.53	0.06	
	Male	157	52.82	27.33			
Self-compassion	Female	283	80.36	15.42	*t*(461) = − 3.38	< 0.01	− 0.06
	Male	180	85.12	13.67			

### Correlations

Pearson correlations revealed a significant positive relationship between PTS and PTG (r = 0.32, p < 0.001), indicating that individuals reporting higher levels of PTS also reported greater PTG. Additionally, PTS was significantly negatively correlated with self-compassion (r = − 0.42, p < 0.001), suggesting that individuals with higher PTS tended to report lower levels of self-compassion. The correlation between PTG and self-compassion was not statistically significant (r = 0.04, p = 0.42).

### Moderation analysis

A simple moderation analysis, using PROCESS 3.3 with 5000 bootstrap samples and 95% confidence intervals, was conducted to investigate the moderation effect of self-compassion on the relationship between PTS (IV) and PTG (DV). Predictors were centred to correct for multicollinearity and only participants who reported exposure to at least one traumatic event were included (n = 413).

**Fig. 2:**
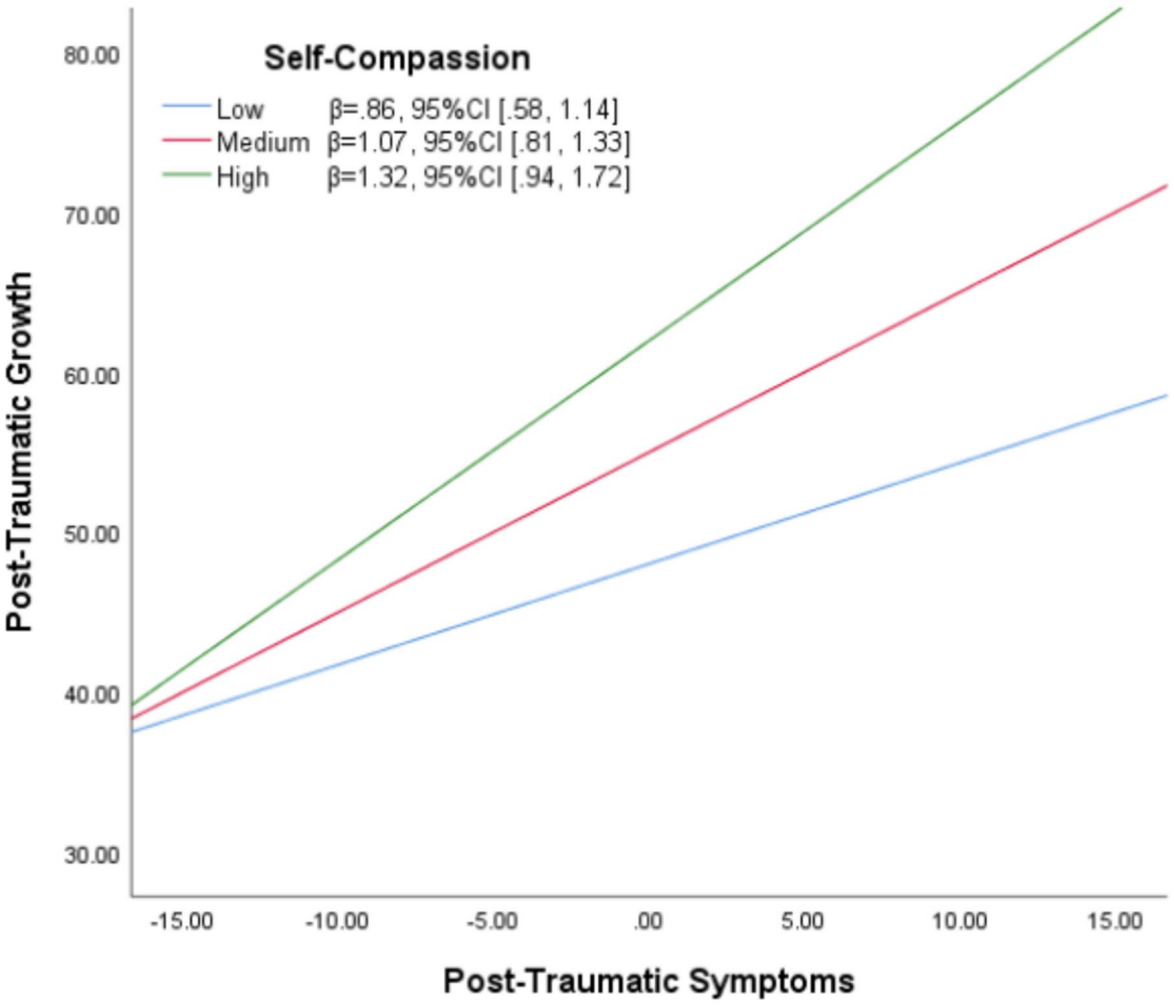
Interaction plot showing the moderation effect of self-compassion on the effects of posttraumatic symptoms on posttraumatic growth.

The analysis revealed a significant interaction effect of PTS and self-compassion on PTG (b = 0.02, 95% CI [0.02, 0.03], p < 0.001), accounting for 16% of the variance in PTG (F(3, 342) = 22.29, p < 0.001). The interaction plot (Fig. 2) illustrated that at higher levels of self-compassion, the positive relationship between PTS and PTG was strengthened. At higher levels of self-compassion, the positive relationship between post-traumatic stress (PTS) and post-traumatic growth (PTG) was strengthened. Specifically, to determine these effects, self-compassion was assessed at three levels: one standard deviation above the mean (high), at the mean (medium), and one standard deviation below the mean (low). Individuals reporting high levels of both PTS and self-compassion demonstrated significantly higher PTG (β = 1.32, 95% CI [0.94, 1.72]) compared to those with medium levels (β = 1.07, 95% CI [0.81, 1.33]) or low levels of both PTS and self-compassion (β = 0.86, 95% CI [0.58, 1.14]).

## Discussion

This study examined how self-compassion influences the relationship between posttraumatic symptoms (PTS) and posttraumatic growth (PTG) among adult trauma survivors. The study highlights a correlation between self-compassion and recovery from trauma, suggesting its potential importance in promoting psychological adaptation and development. This study builds on previous research supporting the notion that self-compassion plays an important role in promoting positive psychological change after a traumatic event^2,32^.

An important finding of the current study is that people who display higher levels of self-compassion tend to experience stronger PTG, even in situations where they experience high levels of PTS. This is consistent with Tedeschi and Calhoun’s^7^ model of PTG, which emphasizes the importance of cognitive processes, such as reappraisal and meaning making, in the recovery process after trauma. Self-compassion is thought to enhance these processes by encouraging a non-judgemental and emotionally regulated response to trauma, which in turn supports psychological development^24,25^.

The moderation analysis revealed that self-compassion enhances the positive correlation between post-traumatic symptoms (PTS) and post-traumatic growth (PTG), aligning with Kleim and Ehlers’s^4^ findings that moderate levels of PTSD correlate with increased PTG. Self-compassion serves as a buffer against the adverse effects of trauma, aiding individuals in finding meaning and personal growth despite distressing experiences^7,24,25^. It not only alleviates the negative impacts of PTS but also increases the potential for PTG, particularly for individuals with high levels of both PTS and self-compassion, who report stronger PTG compared to those with lower self-compassion. By enabling individuals to adopt a broader perspective, reduce self-criticism, and enhance emotion regulation, self-compassion fosters the psychological conditions necessary for growth despite severe stress. This suggests that interventions promoting self-compassion, such as compassion-focused therapy and mindful self-compassion training, may be particularly effective for those with high PTS, fostering resilience, emotional development, and psychological well-being^56,57^. The significant moderating role of self-compassion underscores its importance in trauma recovery interventions^58^.

Consistent with previous research, this study found significant gender differences in PTS and self-compassion^59,60^, although the effect sizes were rather small. Women demonstrated higher levels of PTS and less self-compassion compared to men. This is consistent with previous research indicating that women are more likely to experience increased psychological distress following trauma and may have lower levels of self-compassion due to societal expectations and gender norms^46,60,61^. Socialisation processes often encourage women to prioritise caregiving and victimisation, which may explain their relatively lower levels of self-compassion^45,61^. No significant gender differences in PTG were observed, indicating that both men and women are capable of experiencing personal growth after a traumatic event, although the specific processes may differ. This finding contrasts with meta-analytical findings based on 70 studies that report small to moderate gender differences in PTG^62^. This lack of gender differences in PTG might be due to cultural differences or other sample characteristics. Since the study was conducted exclusively in Cyprus, and cross-cultural comparisons were not possible, it is challenging to determine why gender differences were not observed.

Individuals who reported high levels of both PTS and self-compassion experienced stronger PTG compared to those who reported lower levels of self-compassion. This suggests that self-compassion played a significant role in moderating the relationship between PTS and PTG. This finding suggests that self-compassion may enhance emotional resilience and development by promoting effective coping mechanisms and emotional control following a traumatic event^24,25,57^. Findings highlight the importance of self-compassion as a mitigating factor in trauma recovery interventions^58^.

### Practical implications

The results of this study have significant implications for clinical practice both clinical practice and public health strategies. First, self-compassion’s moderation of the relationship between PTS and PTG suggests that trauma recovery programs could benefit from self-compassion-based interventions. Interventions such as compassion-focused therapy^56^ and mindful self-compassion training^57^ can be particularly effective in enhancing trauma survivors’ capacity to process distress and foster psychological growth. By cultivating self-compassion, physicians and psychologists can help survivors not only alleviate suffering, but also promote good psychological development, thus promoting a more holistic trauma healing process. Second, the gender differences observed in self-compassion and PTS suggest the importance of tailoring interventions to address unique needs based on gender. Third, the findings highlight the potential role of self-compassion in fostering resilience among individuals in high-risk professions, such as healthcare workers, first responders, and military personnel, who frequently face traumatic events. Training programs that focus on developing self-compassion can equip these individuals with tools to manage PTS while promoting long-term psychological well-being and growth. Fourth, self-compassion training in educational settings such as schools and in community centers could help individuals to better cope with traumatic experience and foster a culture of resilience. Finally, policymakers and mental health organizations could consider funding and promoting large-scale programs focused on self-compassion to address trauma recovery on a broader scale. Given the growing body of evidence supporting its efficacy, self-compassion training has the potential to significantly reduce the societal burden of trauma-related mental health challenges, improving both individual and community outcomes.

### Limitations

This study has some limitations. The reliance on self-report measures can introduce biases, such as the tendency to provide socially desirable responses and potential misinformation. Including observational or physiological data in future studies may enhance the validity of the results. Additionally, the current study employed a cross-sectional design, which cannot establish true causality or track changes over time. Longitudinal studies could provide valuable insights into the development of PTS, PTG, and self-compassion throughout the trauma healing process.

The number of participants who reported experiencing a traumatic event was lower than the target sample size estimated in the power analysis. As a result, the study’s statistical power was marginally below the desired threshold, potentially reducing its ability to detect significant effects or associations. This limitation should be taken into account when interpreting the results. The geographic and cultural homogeneity of the sample also limits the generalisability of the results to the wider population. Future research should aim to replicate these findings in diverse cultural settings and examine additional factors that may influence the relationship between PTS post-traumatic growth, and self-compassion.

## Conclusions

This research contributes to the expanding understanding of the psychological processes that foster resilience and growth following trauma. The study underscores the importance of self-compassion in moderating the relationship between PTS and PTG, suggesting that self-compassion plays a crucial role in the trauma healing process. Future research should aim to replicate these findings in diverse populations and identify additional factors that influence the relationship between trauma symptoms, self-compassion, and personal growth. This will ultimately enhance the understanding of trauma recovery and aid in the development of more effective treatment approaches.

## Data Availability

Data available at: 10.6084/m9.figshare.27931476.
